# The C-terminus of PufX plays a key role in dimerisation and assembly of the reaction center light-harvesting 1 complex from *Rhodobacter sphaeroides*

**DOI:** 10.1016/j.bbabio.2017.06.001

**Published:** 2017-09

**Authors:** Pu Qian, Elizabeth C. Martin, Irene W. Ng, C. Neil Hunter

**Affiliations:** Department of Molecular Biology and Biotechnology, University of Sheffield, Sheffield S10 2TN, United Kingdom

**Keywords:** AFM, atomic force microscopy, BChl(s), bacteriochlorophyll(s), B850, bacteriochlorophyll in light harvesting 2 complex with maximal absorption at 850 nm, B875, bacteriochlorophyll in light harvesting 1 complex with maximal absorption at 875 nm, EDTA, ethylenediamine tetraacetic acid, HEPES, *N*-2-hydroxyethylpiperazine-*N*′-2-ethanesulfonic acid, ICM, intracytoplasmic membrane, LH1, light-harvesting 1 complex, LH2, light-harvesting 2 complex, *Rba.*, *Rhodobacter*, RC, reaction center, RC-LH1, reaction center-light-harvesting 1 complex, *Rsp*., *Rhodospirillum*, *Rps.*, *Rhodopseudomonas*, TEM, transmission electron microscopy, FSC, Fourier shell correlation, UPB, upper pigmented band, 2-D, two-dimensional, 3-D, three-dimensional, β-DDM, β-dodecylmaltoglucoside, Bacterial photosynthesis, Reaction centres, Light-harvesting core complex, PufX, Single particle analysis, *Rhodobacter sphaeroides*, Electron microscopy, Quinones

## Abstract

In bacterial photosynthesis reaction center-light-harvesting 1 (RC-LH1) complexes trap absorbed solar energy by generating a charge separated state. Subsequent electron and proton transfers form a quinol, destined to diffuse to the cytochrome *bc*_1_ complex. In bacteria such as *Rhodobacter* (*Rba.*) *sphaeroides* and *Rba. capsulatus* the PufX polypeptide creates a channel for quinone/quinol traffic across the LH1 complex that surrounds the RC, and it is therefore essential for photosynthetic growth. PufX also plays a key role in dimerization of the RC-LH1-PufX core complex, and the structure of the *Rba. sphaeroides* complex shows that the PufX C-terminus, particularly the region from X49-X53, likely mediates association of core monomers. To investigate this putative interaction we analysed mutations PufX R49L, PufX R53L, PufX R49/53L and PufX G52L by measuring photosynthetic growth, fractionation of detergent-solubilised membranes, formation of 2-D crystals and electron microscopy. We show that these mutations do not affect assembly of PufX within the core or photosynthetic growth but they do prevent dimerization, consistent with predictions from the RC-LH1-PufX structure. We obtained low resolution structures of monomeric core complexes with and without PufX, using electron microscopy of negatively stained single particles and 3D reconstruction; the monomeric complex with PufX corresponds to one half of the dimer structure whereas LH1 completely encloses the RC if the gene encoding PufX is deleted. On the basis of the insights gained from these mutagenesis and structural analyses we propose a sequence for assembly of the dimeric RC-LH1-PufX complex.

## 1 Introduction

Purple phototrophic bacteria provide an ideal model for studying the assembly and function of light harvesting-reaction centre (RC) complexes. In all characterized purple bacteria, a light-harvesting complex 1 (LH1) surrounds the RC in a fixed stoichiometry, forming a RC-LH1 core complex [1]. In many bacteria peripheral antenna light-harvesting LH2 complexes [2] funnel excitation energy to the RC-LH1 complex, which elicits a charge separation in the RC. The transfer of excitations from the antenna to the reaction center is the rate-limiting step at this stage of photosynthesis, and it is the major single influence on the overall trapping time [3], [4], [5], [6], [7]. Electron and proton transfers in the RC culminate in the formation of quinol at the RC Q_B_ site, which must leave the complex by traversing the LH1 ring so it can migrate within the lipid bilayer membrane to the nearby cytochrome *bc*_1_ complex [8], [9]. Here, the quinol is oxidised to quinone, and a transmembrane proton gradient is formed [10]. Quinones shuttle back to the RC Q_B_ site, again through the portal in LH1 provided by PufX.

**Fig. 1:**
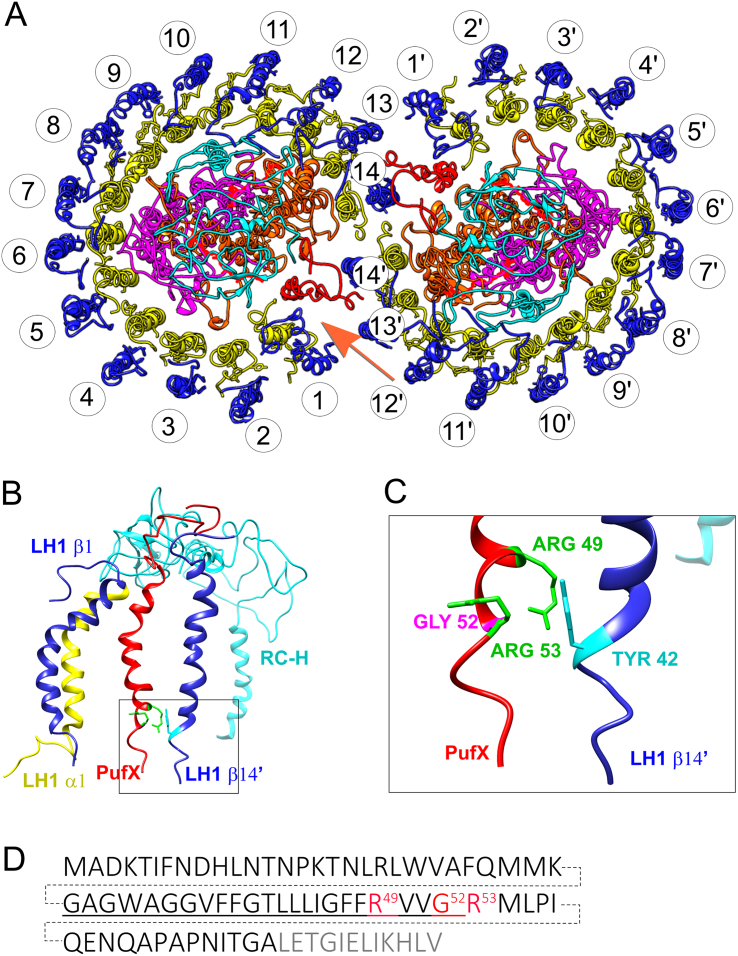
Interactions involving PufX in the dimeric RC-LH1-PufX complex of *Rba. sphaeroides*. A, The cytoplasmic side of the RC-LH1-PufX complex viewed perpendicular to the membrane with LH1β in blue, LH1α in yellow, PufX in red, RC-H in cyan, RC-M in magenta and RC-L in brown. BChl pigments have been omitted for clarity. LH1 αβ subunits are numbered 1–14 on one half of the dimer, 1′–14′ on the other half. The orange arrow indicates the view of the complex in the plane of the membrane used to visualise PufX interactions in more detail in other panels. B, Enlarged C-terminal regions of PufX, RC-H, LH1 α1 and LH1 β14′ viewed in the plane of the membrane. C, Zoomed in image showing the locations of residues altered in this study. D, The primary sequence of *Rba. sphaeroides* PufX, with the residues altered in the present study highlighted in red. The numbering starts at the N-terminal Met; this residue and the C-terminal residues in gray are removed by processing. The residues corresponding to the transmembrane are underlined.

Cryo-electron microscopy, single particle analysis and X-ray crystallography of the ~ 521 kDa RC-LH1-PufX dimer complex [11], [12], [13], comprising 64 polypeptides, 80 transmembrane helices and 128 cofactors, showed that 28 LH1 αβ subunits form an S-shaped assembly that intertwines round two RCs (Fig. 1A). In each half of the complex a PufX polypeptide, located adjacent to the RC Q_B_ site, provides a pore for quinone exchange by preventing complete encirclement of the RC [13]. The N-terminal domain of PufX, on the cytoplasmic side of the membrane [14], forms a close association with the extrinsic domain of the RC-H subunit [13], [15]. As also shown by NMR [15], [16], the rest of PufX forms a bent transmembrane helix. The loss of the quinone portal, through deletion of the *pufX* gene, prevents photosynthetic growth in *Rba. sphaeroides* and *Rba. capsulatus* [17], [18], [19], [20] because the space in the LH1 ring occupied by PufX is replaced by additional LH1 αβ subunits that surround the RC and slow down quinone migration significantly [21], [22], [23]. Suppressor mutations that relieve this blockage do so by impairing LH1 assembly and opening gaps in the LH1 ring [24], [25]. Other LH1 mutations that prevent encirclement of the RC or impair carotenoid binding also compensate for the loss of PufX [26], [27].

PufX also determines the dimerization of the RC-LH1-PufX complex, which in turn imposes curvature on the membrane because the two halves of the RC-LH1-PufX structure incline towards each other at a shallow angle [12], [13], [28]. Deletion of the *pufX* gene, or mutagenesis of the PufX N-terminus, produces flattened membranes in the absence of the other curvature-inducing complex, LH2 [15], [29], [30]. The spherical intracytoplasmic vesicles (‘chromatophores’) of *Rba. sphaeroides* and *Rba. capsulatus* are a consequence of this curvature, as is the partitioning of complexes into zones enriched in either LH2 or RC-LH1-PufX complexes [8], [31], [32], [33], [34], [35]. Given that the assembly, function and morphology of these vesicles are heavily influenced by PufX, we set out to examine the interactions of PufX that influence core complex dimerization. The importance of the PufX N-terminus is well established; it binds to the extrinsic domain of the RC-H subunit, and removal of 12 or more residues from the PufX N-terminus abolishes dimerization of the core complex [15], [36]. Interactions at the PufX C-terminus are less well defined, but it is known that truncations of 7,11 and 15 residues from the C-terminus of the PufX abolishes dimerisation of the core complex [36]. The 8.0 Å resolution of the RC-LH1-PufX structure [13] is not sufficient for precise assignment of the protein–protein contacts at the PufX C-terminus, although it was possible to identify a close association between the PufX C-terminal region and LH1β14′ polypeptide on the other half of the complex (Fig. 1B). Fig. 1C shows that the C-terminal residues X49-X53 and Tyr42 of LH1-β14′ could be involved in stabilising the interface between the two halves of the RC-LH1-PufX dimer. In this study we test this structural model by performing site-directed mutagenesis of the PufX residues R49, G52 and R53 as well as Y42 (+ 4 in relation to the conserved BChl ligand His0) of LH1 β14, β14′, analyse the resulting core complexes, compare 3D reconstituted models of the core complexes with and without PufX, and we propose an assembly sequence for the dimeric core complex from *Rba. sphaeroides*.

## 2 Materials and methods

### 2.1 *Rhodobacter sphaeroides* strains

All strains used in this work are constructed by direct modification of the *Rba. sphaeroides* genome as described [37]. These include PufX-minus (PufX^−^), PufX R49L, PufX R53L, PufX R49/53L, PufX G52L, PufB Y43F, PufB Y43L and PufBA (LH1)-minus. All mutations were confirmed by sequence analysis.

### 2.2 Cell cultures

All cells, except for the PufX^−^ mutant, were grown photosynthetically in M22 medium under a light intensity of 150 μmol photons s^− 1^ m^− 2^ using Osram 116 W halogen bulbs. When the culture reached an absorbance of 1.6 at 680 nm cells were harvested by centrifugation at 3290*g* for 30 min. The cells were suspended in working buffer (20 mM HEPES, pH 7.8) and stored at − 20 °C. For measurement of kinetics of photosynthetic growth various *Rba. sphaeroides* strains were inoculated into a glass tubes (1.5 × 13 cm) fully filled with M22 medium. Each inoculum was taken from a 10 ml starter culture grown under semi-aerobic conditions, and the starting value of the 680 nm absorbance of each culture was adjusted to 0.1. Triplicates of each strain were stirred, and illuminated using the same light source as for large scale cultures. Growth was monitored using a WPA Colourwave CO7000 Medical Colorimeter equipped with a 680 nm filter.

### 2.3 Purification of core complexes

Cells were washed with working buffer. Washed cells were passed through French Press three times under a pressure of 18,000 psi. The broken cell suspension was loaded onto a two-step sucrose gradient (15%-40% w/w sucrose in working buffer) and centrifuged for 5 h at 100,000*g* (27,000 rpm, Beckman SW30 rotor). Intracytoplasmic membranes were collected from the 15/40% interface, diluted three-fold with working buffer and pelleted for 1 h at 235,000*g* (45,000 rpm, Beckman Type 45Ti rotor). The pellet was re-suspended in working buffer, and its OD was adjusted to ~ 100 at 850 nm. The photosynthetic membrane was adjusted to OD 60 at 850 nm, and solubilized in the dark using 3% (w/w) β-DDM for 30 min with stirring. Unsolubilised material was removed by centrifugation for 1 h at 211,000*g* (48,000 rpm, Beckman 70.1Ti rotor). The clear supernatant was loaded on to a five-step discontinuous sucrose gradient (20%, 21.25%, 22.5%, 23.75% and 25% sucrose in working buffer with 0.03% β-DDM), and was centrifuged for 16 h at 125,000*g* (27,000 rpm, Beckman SW41 rotor). Core complex bands were harvested and applied to an ion exchange column (DEAE-Sepharose, Sigma) equilibrated in working buffer containing 0.03% β-DDM. Core complexes, which eluted at 250 mM NaCl, were collected and concentrated then purified further on a Superdex 200 gel filtration column pre-equilibrated in working buffer containing 0.03% β-DDM and 100 mM NaCl. Fractions with an A_880_/A_270_ absorbance ratio higher than 1.7 were pooled and concentrated.

### 2.4 Immunodetection of PufX

The NuPAGE pre-cast gel system (Invitrogen, USA) was used to separate polypeptides by SDS-polyacrylamide gel electrophoresis. Purified membranes were incubated at 70 °C for 10 min in NuPAGE LDS sample buffer (Invitrogen, USA). Immunodetection of the PufX protein was performed using enhanced chemiluminescence detection regents supplied by Amersham (ECL Detection System, USA).

### 2.5 Dialysis of complexes to form 2D crystals

The protein concentration for 2-D crystallization was adjusted to 0.5 mg/ml, then mixed with a 4 mg/ml extract of *E. coli* lipids (Avanti Polar Lipids, USA), to give a lipid/protein ratio from 0.3 to 0.6 (w/w). Each 100 μl aliquot of these mixtures was dialysed against buffer solution (150 mM NaCl, 20 mM HEPES, 0.01% Na_3_N, pH 7.5) at 20 °C for 10 days using a home-made continuous flow device [38].

### 2.6 Electron microscopy

The purified core complexes from PufX R49/53L and PufX^−^ mutants were used for 3D EM single particle reconstruction. Protein solutions were diluted to an A_874_ of 1.0 prior to preparation of TEM grids and negative staining. Five μl protein solution were applied on the surface of a carbon coated 400 mesh Cu grid, which was glow discharged for 30 s before use. Excess protein solution was blotted away by touching the edge of the grid with filter paper. The grid was then washed twice with water, stained with 0.75% uranyl formate for 30 s, then dried in air. All images were recorded at room temperature using a Philips CM-100 microscope equipped 1 K × 1 K Gatan Multiscan 794 CCD camera. Magnification was set at 61,000 X corresponding to 3.93 Å per pixel at the specimen level. The defocus value was varied between ~ 0.5 to 1.5 μm.

### 2.7 Image processing

All images showing non-drifting, well stained particles and with suitable defocus value were selected for particle picking. A 64 × 64 pixel box, corresponding to 25 × 25 nm at the specimen level, was used for particle picking by the use of e2boxer in EMAN2 [39]. In total, 13,030 core complex particles were collected for the PufX R49/53L mutant. After CTF collection, a data subset consisting of 5022 PufX R49/53L mutant core complexes was compiled for reference-free 2D classification, from which an initial 3D model of the core complex was calculated.

The initial 3D model together with the subset of data was used for the first stage of 3D reconstruction. A stable and improved 3D model from the first stage calculation was applied to the full dataset in the second stage of refinement. The refinement was iterated until calculation of the structural resolution indicated no improvement of the 3D model reconstruction. A similar method was applied to the 3D reconstruction of the core complex from the PufX^−^ mutant, which used 10,866 collected particles.

### 2.8 Prediction of the conformation of the PufX C-terminus

Structural predictions for mutant PufX polypeptides were calculated using the method of iterative threading assembly refinement (I-TASSER) [40] and performed using the on-line server provided by the I-TASSER authors (http://zhanglab.ccmb.med.umich.edu/I-TASSER/).

## 3 Results and discussion

### 3.1 Photosynthetic growth curves of five different *Rba. sphaeroides* strains: PufX-minus, PufX R49L, PufX R53L, PufX R49/53L and wild-type

**Fig. 2:**
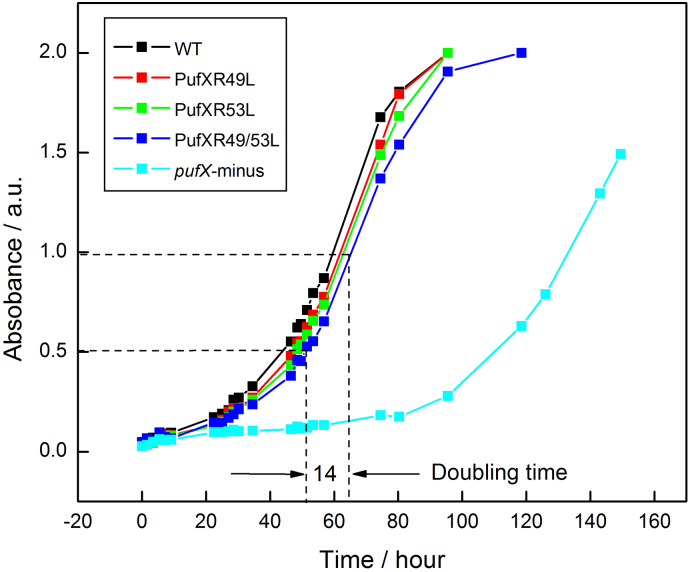
Photosynthetic growth curves for five different *Rba. sphaeroides* strains. The colour coding for each strain is displayed in the inset. Each data point is the average of three biological replicates. For clarity the doubling time of the PufX R49/53L mutant only is indicated using dashed lines.; the variations among these cultures are presented in Fig. S1, which also shows growth data displayed as semilogarithmic plots.

Wild-type and mutant strains of *Rba. sphaeroides* were grown photoheterotropically as described in Materials and Methods. Fig. 2 shows that exponential growth of the PufX-containing mutants PufX R49L, PufX R53L and PufX R49/53 commenced after a ~ 15 h lag phase due to an adaption of the cells from semi-aerobic to anaerobic photosynthetic growth, consistent with previous work on PufX mutants [15], [41]. These experiments were performed with three biological replicates and showed good reproducibility (see Fig. S1B–D) and photosynthetic growth of the PufX-containing mutants almost matched that of the wild-type (Fig. S1A). The overall kinetics of growth of PufX-containing strains are similar with a doubling time of 14 h, but growth of the PufX^−^ mutant only occurred after ~ 80 h with a wide variation of the growth curves among the three parallel cultures (Fig. 2, Fig. S1E, Fig. S1F). This unpredictable growth of PufX-deficient strains, also observed previously [18], [24] has been proposed to arise from suppressor mutations [20], [24] that limit assembly of the LH1 antenna, thereby creating gaps in the LH1 that allow quinone/quinol traffic. The maintenance of near-wild-type growth rates in PufX R49L, PufX R53L, PufX R49/53L strains shows that these alterations have likely not affected incorporation of PufX into the RC-LH1 core complex.

### 3.2 The effects of altering C-terminal residues of PufX on core dimerisation – analysis on sucrose density gradients

**Fig. 3:**
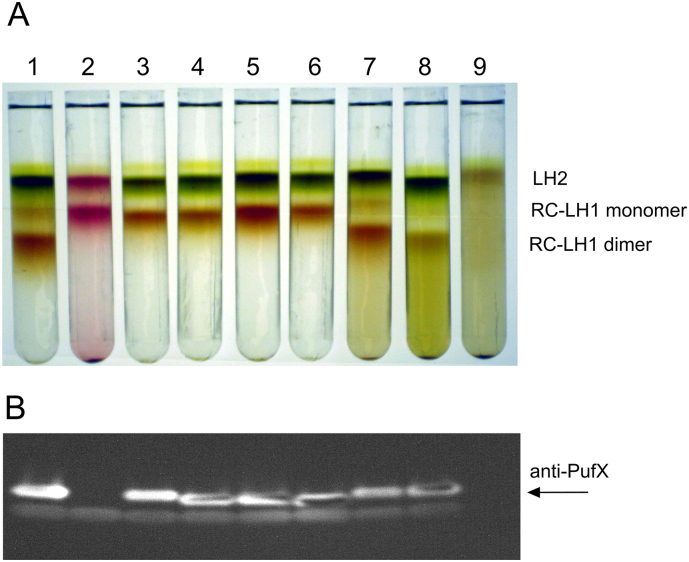
Separation and analysis of monomer and dimer core complexes. (A) Fractionation of detergent-solubilised LH2, core monomer and dimer complexes from wild-type and mutant strains on sucrose density gradients. 1—wild-type; 2—PufX^−^; 3—PufX R49L; 4—PufX R53L; 5—PufX R49/53L; 6—PufX G52L; 7—PufB Y43F; 8—PufB Y43L; 9—ΔPufBA. (B) Immunoblot of photosynthetic membrane samples corresponding to those in the upper panel, probed with antibodies to PufX.

RC-LH1-PufX complexes were extracted from photosynthetic membranes of wild-type and mutant cells using β-DDM, and fractionated by sucrose gradient centrifugation (Fig. 3A). The three major bands, aside from the free pigment, were assigned from top to bottom as LH2, core monomer and core dimer. All three fractions are represented in the wild-type, whereas the Δ*pufBA* mutant only has the LH2 band as expected. LH2 and monomeric core complexes are found in all five PufX mutants, PufX^−^, PufX R49L; PufX R53L; PufX R49/53L and PufX G52L, although dimeric core complexes were found in fractionated membranes from *pufB* mutants with a normal PufX, i.e., PufB Y43F, PufB Y43L. We note that complexes in the PufX^−^ (tube 2) have a different colour compared with the other strains because of the conversion of the carotenoid spheroidene to spheroidenone under the semi-aerobic culture conditions required for this non-photosynthetic mutant [23], [34]. The immunoblot in Fig. 3B shows that PufX is present in photosynthetic membranes of PufX R49L, PufX R53L, PufX R49/53L, PufX G52L, PufB Y42F and PufB Y42L mutants, with no PufX detected in ΔPufX and ΔPufBA mutants. Thus, the effects of site directed mutations in Fig. 3, lanes 3–6 cannot be ascribed to a lack of PufX. It is clear that the conserved Arg49 and Arg53 residues in the PufX C-terminal region are determinants of core complex dimerization; alteration of either residue to Leu abolishes dimer formation (Fig. 3, lanes 3, 4). Changing the Gly at position X52 to Leu also prevents dimer formation (Fig. 3, lane 6), which underlines the importance of this region of PufX. Alteration of LH1β Trp43 to Phe or Leu, on the other hand, has no such effect, implying that any interaction between the PufX on one half of the dimer and LH1β14′ on the other half is not through a hydrogen bond. Thus, the alterations at positions X52 and X53 destabilise the association with LH1β14′ on the other half of the complex (see Fig. 1B, C), possibly by changing the conformation of the PufX C-terminal domain. This point is examined in Section 3.6 and Fig. S4. A more precise assignment of the native monomer-monomer interaction requires a higher resolution 3D structure of dimeric core complex from *Rba. sphaeroides*.

### 3.3 Purification of core complexes from PufX^−^, PufX R49/53L and DD13G[pRKEH] mutants

Following separation on sucrose density gradients, the monomer bands from the PufX^−^ and PufX R49/53L mutants were collected and further purified as described in Materials and methods. For comparison, we also purified the RC-LH1-PufX dimer from strain DD13G[pRKEH] [13]. The purity of the core complex preparations was monitored by their A_874_/A_280_ absorbance ratio, which reached 1.87, 1.84 and 2.0 respectively in the best fraction from gel filtration chromatography. Fig. S2 shows an analysis of these preparations by SDS-PAGE; in all samples there are three RC bands labelled H, M and L and two bands from the LH1 α and β polypeptides. A weak band corresponding to PufX can be seen just above LH1 α in lanes 2 (WT core complex) and 4 (PufX R49/53L core complex), but not in lane 3 for the complex from the PufX^−^ mutant, as expected.

**Fig. 4:**
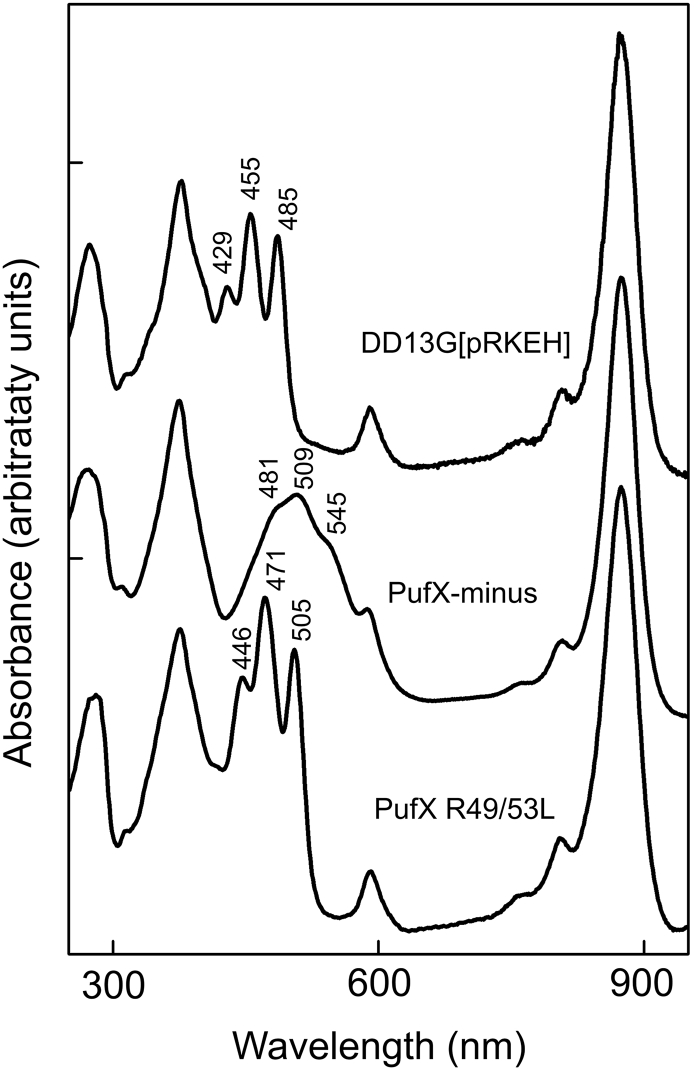
Absorption spectra of purified core complexes from DD13G[pRKEH], PufX^−^ and PufX R49/53L mutants. The spectra are normalized to the LH1 Qy peak of BChl *a* in the core complexes at 874 nm.

The different carotenoid contents of the complexes are manifested in the 350–550 nm region of the absorption spectra in Fig. 4. The carotenoid in the core dimer purified from strain DD13G[pRKEH] is neurosporene, with absorption maxima at 429, 455 and 486 nm, whereas the RC-LH1-PufX monomer core complex from the photosynthetically grown PufX R49/53L mutant has spheroidene (maxima at 446, 471 and 505 nm) as the major carotenoid. Finally the complex from the PufX^−^ mutant, which was grown semi-aerobically because of its inability to photosynthesise, has the broad absorption and maxima (481, 509 and 545 nm) of spheroidenone.

The A_874_/A_800_ absorbance ratio, taken from the spectrum in Fig. 4, was used to estimate the LH1: RC stoichiometry; the value of 4.7 for the DD13G[pRKEH] core dimer and for the PufX R49/53L complex shows that the purified monomer from the PufX R49/53L mutant corresponds to one half of the dimer in wild-type *Rba. sphaeroides*. However, the ratio of 5.9 for the PufX^−^ core complex indicates that removal of the PufX polypeptide likely allows the assembly of additional LH1 subunits that completely encircle the RC [37]. This point is examined further in 3.4, 3.5.

### 3.4 The effects of altering C-terminal residues of PufX on core dimerisation – supramolecular arrangement of DD13G[pRKEH] and PufX R49/53L mutant core complexes in 2D crystals

**Fig. 5:**
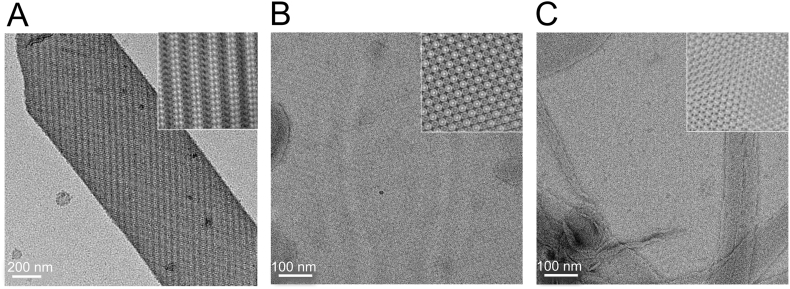
Negatively stained 2D crystals of *Rba. sphaeroides* core complexes. A, DD13G[pRKEH] complexes. B, PufX R49/53L. C, PufX^−^ complexes. The inserts are Fourier filtered images of the electron micrographs.

We assessed the influence of the PufX R49/53L alteration on the inter-complex associations that drive formation of ordered arrays of complexes. Purified *DD13G[pRKEH]* and mutant core complexes were mixed with lipids in buffer solution and dialysed against detergent-free buffer solution to form 2-D crystals. Fig. 5A shows that the DD13G[pRKEH] core dimer forms tubular crystals, as found previously [12]; flattening on the carbon film support yields two crystalline molecular layers of negatively stained dimer core complexes. The Fourier filtered image in the inset to Fig. 5A clearly shows linear arrays of core dimers, which adopt an up-down-up orientation visible as alternating dark/light stripes. Fig. 5B shows the 2D crystals formed by the PufX^−^ negative control; the absence of PufX abolishes dimer formation and instead of tubes we now see highly ordered sheets of RC-LH1 monomers. PufX-containing but monomeric cores of the PufX R49/53L mutant (Fig. 5C) are clearly unable to form dimers, and therefore the dimer–dimer associations found in Fig. 5A. Instead these cores form ordered hexameric arrays, rather than the tetrameric arrays found in the absence of PufX (Fig. 5B).

### 3.5 3D reconstruction of monomeric core complexes from PufX^−^ and PufX R49/53L mutants

**Fig. 6:**
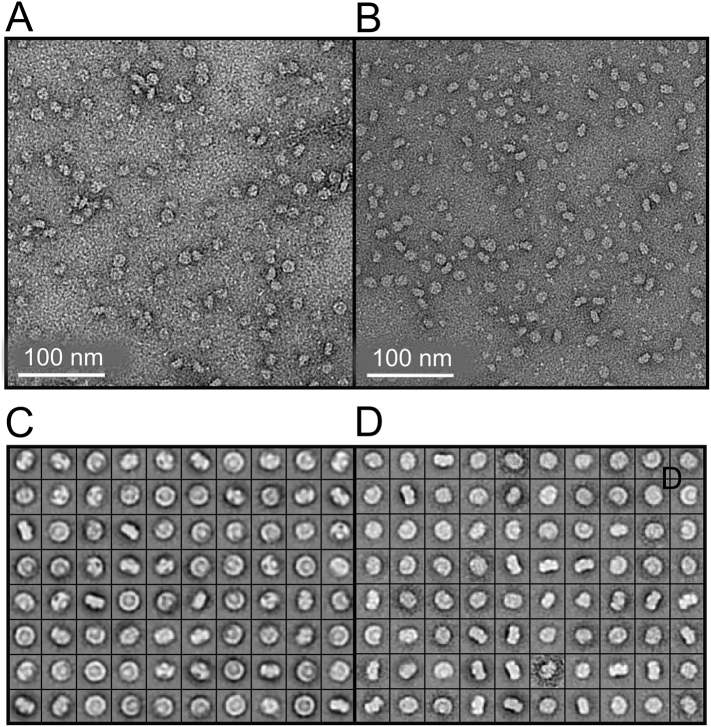
Electron micrographs of negatively stained core complexes. A, PufX^−^ core complexes. B, PufX R49/53L complexes. C, D Classified 2D projections of the core complexes from PufX^−^ and PufX R49/53L core complexes respectively. Box size = 25 × 25 nm.

The purified core complexes from PufX^−^ and PufX R49/53L mutants were absorbed on an EM grid coated with carbon film, negatively stained and imaged using TEM. Under negative stain conditions and at 60,000 x magnification, core complexes from PufX^−^ and PufX R49/53L mutants can be seen clearly in Fig. 6A and B; 5022 and 4342 single particles respectively were picked and assigned into 80 classes, which are shown in Fig. 6C and D. These classes represent a series of tilted angles of the PufX^−^ and PufX R49/53L complexes, which are monomeric as expected from the analyses in Fig. 3, Fig. 5.These classes are used for creation of initial 3D models of the core complexes.

**Fig. 7:**
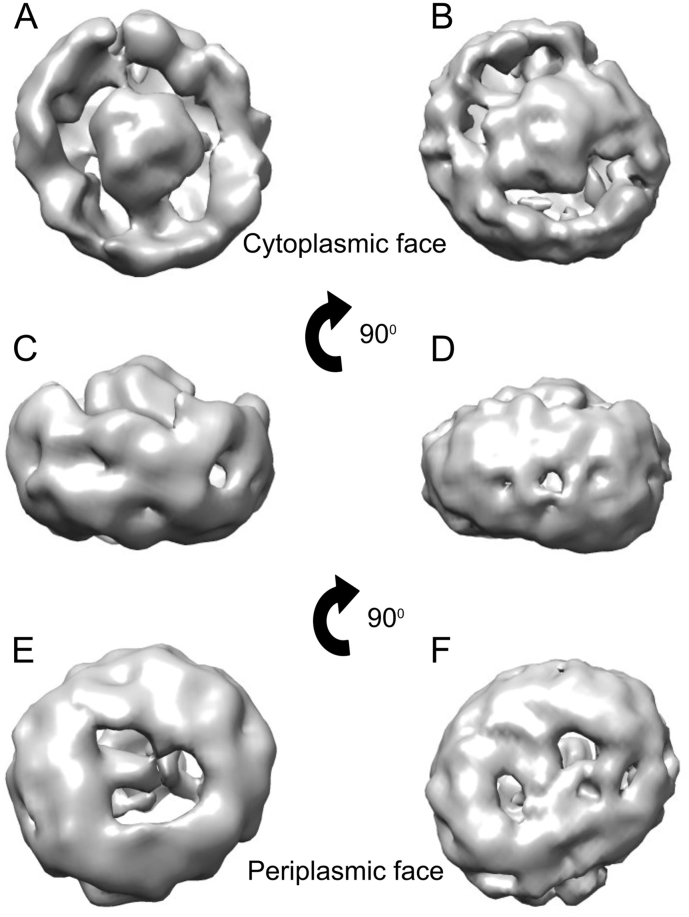
Single particle analysis of negatively stained core monomer complexes with (right) and without (left) PufX. A,B top view from cytoplasmic side; C,D side view with the cytoplasmic side on top; E,F bottom view from periplasmic side. Images were produced using UCSF Chimera [43].

The refined 3D models of the two types of core complex are shown in Fig. 7. The resolutions of 3D models of the core complex from PufX^−^ and PufX R49/53L mutants were calculated to be 18.2 Å and 20.8 Å based on 0.5 Fourier shell correlation (FSC) criteria, respectively (Fig. S3). Although this range of resolution is not high enough to resolve transmembrane helices, and therefore the positions of individual LH1 α/β subunits, the 3D models reveal general structural features of the complexes at sufficient detail to distinguish them from one another. The lower correlation coefficient, 0.78, between these two models indicates that they are two different complexes. Top views of the core complexes from the cytoplasmic side (Fig. 5 panel A; without PufX and B; with PufX) show that in the absence of PufX the LH1 complex encircles the RC to form a complete elliptical ring with a long axis of 14.6 nm and a short axis of 13.7 nm. The structure of the PufX R49/53L mutant complex provides the first view of a monomeric complex containing PufX, which has an elliptical LH1 ring with a long axis of 14.4 nm and a short axis of 12.6 nm. In addition, a continuous gap between LH1 and the RC can be seen from top view of the PufX^−^ mutant core complex, which reflects pooling of the negative stain. In contrast, the close association of the PufX with RC-H in the PufX R49/53L mutant core complex interrupts this RC-LH1 gap and also shortens the short axis of the core complex. The side views in panels C and D show that RC-H protrudes from the cytoplasmic side of the core complexes. The total heights of the complexes measured from top of RC-H to the bottom of the LH1 C-terminus are ~ 10.2 nm, in agreement with measurements from the 3D dimer structure [13] and AFM of purified complexes [42]. The periplasmic side of each complex is shown in Fig. 7E, F.

**Fig. 8:**
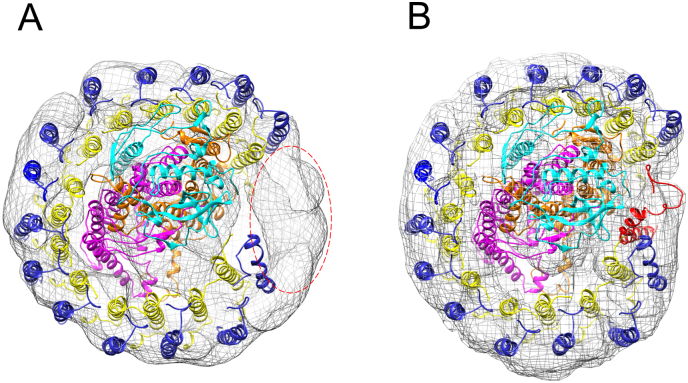
Superposition of EM reconstruction models (in mesh) with the X-ray structure (in ribbon) of one half of the dimeric core complex. A, Half of the RC-LH1-PufX dimer X-ray structure, but with PufX omitted, is modelled into the density for the PufX^−^ complex. An extra density outlined with a red ellipse corresponds to two extra LH1αβ subunits. B, Half of the RC-LH1-PufX dimer X-ray structure is modelled into the density of the monomeric PufX R49/53L complex. LH1β in blue, LH1α in yellow, PufX in red, RC-H in cyan, RC-M in magenta and RC-L in brown. BChl pigments have been omitted for clarity. The EM models were shrunk 10% to reduce or eliminate the effects caused by detergent and stain on their size [46], [47], [48].

Fig. 8 shows the EM structures in Fig. 7B, D, F viewed from the cytoplasmic side; half of the dimeric RC-LH1-PufX structure from wild-type *Rba. sphaeroides* has been superimposed onto the structural models for the PufX^−^ and PufX R49/53L core complexes (Fig. 8A, B, respectively). The RC-LH1-PufX complex, in which the RC is surrounded by 14 LH1 αβBChl_2_Crt_2_ subunits and a PufX polypeptide, does not match the overall shape of the PufX^−^ core complex. Fig. 8A (oval circle) shows a region of unassigned density that can accommodate two extra LH1 αβ sub-units, allowing them to form a complete LH1 ring as found for the naturally PufX^−^ RC-LH1 complexes of *Rhodospirillum* (*Rsp.*) *rubrum* and *T. tepidum* [42], [44], [45]. Thus, in the absence of a PufX polypeptide, the vacancy in the ring is filled by two extra LH1 αβBChl_2_Crt_2_ subunits. The good match between the monomer RC-LH1-PufX complex and the EM model for the PufX R49/53L mutant (Fig. 8B) indicates that the X49L/X53L double mutation has not affected the assembly of this core monomer complex.

### 3.6 Effects of altering the X49-53 region of PufX

The 8.0 Å resolution X-ray structure of the dimeric core complex from *Rba. sphaeroides* [13] indicates a functional role of the C-terminal domain in dimerisation, involving PufX-LH1 β14′ and PufX′-LH1 β14, which we tested using site-directed mutagenesis. C-terminal alteration of R49, G52 or R53 prevents dimer formation but the basis for this effect is unclear. It has been suggested that disruption of the membrane-spanning helix could change native interactions of PufX outside the membrane bilayer [40]. The site-directed mutations we performed are located near the membrane surface on the periplasmic side, and the lack of high resolution 3D structural information for wild-type and mutant complexes prompted a modelling study to find out how site-directed mutations could confer structural changes on PufX. Predicted structures of PufX mutants were aligned against wild-type PufX; for each mutant PufX, the model in green has the lower RMSD value, and the cyan model a slightly higher value (Fig. S4). The N-termini and transmembrane helical regions align reasonably well with the wild-type PufX structure but the orientations of the mutant C-terminal domains differ. These simulations indicate that alterations of PufX in the X49–53 region could perturb the interface with another monomer that stabilises dimer formation. However, changing LH1 β Y42 to F or L, which could have disrupted the interaction between PufX and Tyr 42 from the opposing half of the dimer (Fig. 1), did not affect dimerization (Fig. 3A). This experiment is limited by the lack of specificity of the mutation; all 28 copies of LH1 β in the RC-LH1-PufX dimer are necessarily altered by changing the *pufB* gene and indeed LH1 assembly appears to be affected in the Y42L mutant. Orientations of side chains cannot be resolved in the structural model of the RC-LH1-PufX dimer [13], so the positions of one of the LH1 β Y42 residues close to the opposing PufX could be incorrectly assigned. Nevertheless, the structural model does correctly identify C-terminal residues in PufX that influence dimerization, and the importance of R49, G52 and R53 has been tested and verified by mutagenesis.

### 3.7 Sequence of assembly of dimeric RC-LH1-PufX core complexes

**Fig. 9:**
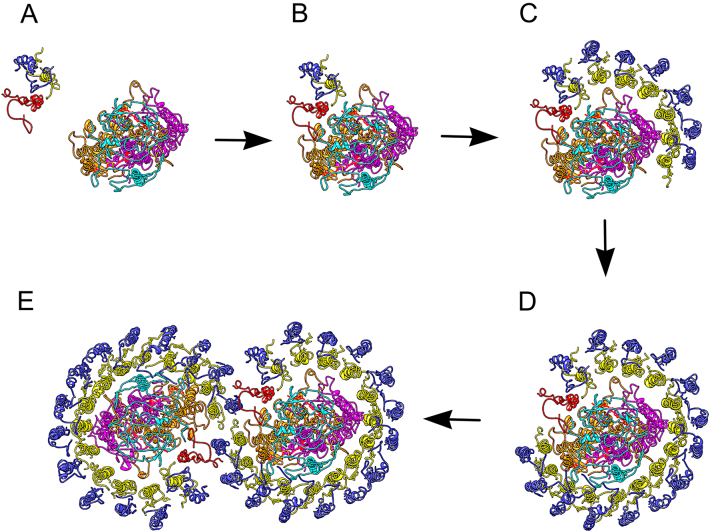
Schematic representation of the assembly of the dimeric core complex of *Rba. sphaeroides*. A,B, The ‘start signal’ for assembly of the RC-LH1-PufX complex is linkage of a PufX + LH1 αβBChl_2_Crt_2_ subcomplex to RC-H, which provides an anchoring point for building the LH1 ring. Subsequent LH1 αβBChl_2_Crt_2_ subunits, ensuring they do not block the RC Q_B_ site. C, Encirclement of the RC proceeds as more LH1 αβBChl_2_Crt_2_ subunits arrive. D, the RC acts as a template for correct sizing of the LH1 ring. This is the end point for some of the core complexes. E, C-terminal contacts between each PufX and its opposing LH1 β14 partner promote dimerization. LH1β is in blue, LH1α in yellow, PufX in red, RC-H in cyan, RC-M in magenta and RC-L in brown. BChl pigments have been omitted for clarity.

The mutations reported in this work prevent the final stage of assembly of the RC-LH1-PufX dimer (Fig. 9), a process proposed to begin with assembly of an LH1 αβBChl_2_Crt_2_-PufX cluster [14]. Inhibition of LH1 reconstitution in vitro by PufX and the tendency of the PufX to associate with the LH1α polypeptide during its purification [49] support this model. The PufX N-terminal domain docks onto the RC-H C-terminal region which ensures that PufX and its attendant LH1αβBChl_2_Crt_2_ subunit are correctly positioned to initiate the subsequent encirclement of the RC. This PufX-RC-H association creates a pore in the LH1 ring that allows quinone/quinol exchange at the RC Q_B_ site, and is a likely ‘start’ signal for arrival of more LH1αβBChl_2_Crt_2_ units. Once started, this process of encirclement proceeds to completion, with the RC acting as a template [50], [51]. Fractionation of LH1 complexes by lithium dodecyl sulphate polyacrylamide gel electrophoresis forms a ladder of LH1 complexes differing in mass by one LH1α_1_β_1_BChl_2_Crt_2_ unit, which supports the idea of progressive formation of the LH1 ring [52].

The final step, where two monomers associate, is not inevitable and some monomers are always present in wild-type membranes; for example they can account for half of all cores when spheroidenone is the carotenoid [37]. The association of monomers has important consequences for the next step in photosystem assembly, because it creates the local membrane bending [12], [37] that favours the incorporation of LH2 complexes into the intracytoplasmic membrane [32], [37], and association to form extensive light-harvesting LH2 domains [31], [32], [34], [35].

## Conflict of interest

The authors declare no conflict of interest.

## Transparency document

Transparency document. Image 1

## Appendix A Supplementary data

Supplementary figures Image 1

## References

[1] Bullough P.A., Qian P., Hunter C.N., Hunter C.N., Daldal F., Thurnauer M.C., Beatty J.T. (2008). Reaction center-light-harvesting core complexes of purple bacteria. The Purple Phototrophic Bacteria.

[2] Cogdell R.J., Gall A., Kîhler J. (2006). The architecture and function of the light-harvesting apparatus of purple bacteria: from single molecules to in vivo membranes. Q. Rev. Biophys..

[3] Visscher K.J., Bergström H., Sundström V., Hunter C.N., van Grondelle R. (1989). Temperature dependence of energy transfer from the long wavelength antenna Bchl-896 to the reaction center in *Rhodospirillum rubrum*, *Rhodobacter sphaeroides* (w.t. and M21 mutant) from 77 to 177K, studied by picosecond absorption spectroscopy. Photosynth. Res..

[4] Beekman L.M.P., van Mourik F., Jones M.R., Visser H.M., Hunter C.N., van Grondelle R. (1994). Trapping kinetics in mutants of the photosynthetic purple bacterium *Rhodobacter sphaeroides*: the influence of the charge separation rate. Biochemistry.

[5] Sundström V., van Grondelle R., Bergström H., Åkesson E., Gillbro T. (1986). Excitation-energy transport in the bacteriochlorophyll antenna systems of *Rhodospirillum rubrum* and *Rhodobacter sphaeroides*, studied by low-intensity picosecond absorption spectroscopy. Biochim. Biophys. Acta.

[6] Sener M.K., Olsen J.D., Hunter C.N., Schulten K. (2007). Atomic level structural and functional model of a bacterial photosynthetic membrane vesicle. Proc. Natl. Acad. Sci. U. S. A..

[7] Chenchiliyan M., Timpmann K., Jalviste E., Adams P.G., Hunter C.N., Freiberg A. (2016). Dimerization of core complexes as an efficient strategy for light harvesting in *Rhodobacter sphaeroides*. Biochim. Biophys. Acta Bioenerg..

[8] Cartron M.L., Olsen J.D., Sener M., Jackson P.J., Brindley A.A., Qian P., Dickman M.J., Leggett G.J., Schulten K., Hunter C.N. (2014). Integration of energy and electron transfer processes in the photosynthetic membrane of *Rhodobacter sphaeroides*. Biochim. Biophys. Acta Bioenerg..

[9] Lavergne J., Verméglio A., Joliot P., Hunter C.N., Daldal F., Thurnauer M.C., Beatty J.T. (2008). Functional coupling between reaction centers and cytochrome *bc*_1_ complexes. The Purple Phototrophic Bacteria.

[10] Crofts A.R., Meinhardt S.W., Jones K.R., Snozzi M. (1983). The role of the quinone pool in the cyclic electron-transfer chain of *Rhodopseudomonas sphaeroides*. A modified Q-cycle mechanism. Biochim. Biophys. Acta.

[11] Qian P., Hunter C.N., Bullough P.A. (2005). The 8.5 Å projection structure of the core RC-LH1-PufX dimer of *Rhodobacter sphaeroides*. J. Mol. Biol..

[12] Qian P., Bullough P.A., Hunter C.N. (2008). 3-D reconstruction of a membrane-bending complex: the RC-LH1-PufX core dimer of *Rhodobacter sphaeroides*. J. Biol. Chem..

[13] Qian P., Papiz M.Z., Jackson P.J., Brindley A.A., Ng I., Olsen J.D., Dickman M.J., Bullough P.A., Hunter C.N. (2013). The 3-D structure of the *Rhodobacter sphaeroides* RC-LH1-PufX complex: dimerization and quinone channels promoted by PufX. Biochemistry.

[14] Pugh R.J., McGlynn P., Jones M.R., Hunter C.N. (1998). The LH1-RC core complex of *Rhodobacter sphaeroides*: interaction between components, time-dependent assembly, and topology of the PufX protein. Biochim. Biophys. Acta.

[15] Ratcliffe E.C., Tunnicliffe R.B., Ng I.W., Adams P.G., Qian P., Holden-Dye K., Jones M.R., Williamson M.P., Hunter C.N. (2011). Experimental evidence that the membrane-spanning helix of PufX adopts a bent conformation that facilitates dimerisation of the *Rhodobacter sphaeroides* RC-LH1 complex through N-terminal interactions. Biochim. Biophys. Acta.

[16] Tunnicliffe R.B., Ratcliffe E.C., Hunter C.N., Williamson M.P. (2006). The solution structure of the PufX polypeptide from *Rhodobacter sphaeroides*. FEBS Lett..

[17] Klug G., Cohen S.N. (1988). Pleiotropic effects of localized *Rhodobacter capsulatus puf* operon deletions on production of light-absorbing pigment-protein complexes. J. Bacteriol..

[18] Farchaus J.W., Gruenberg H., Oesterhelt D. (1990). Complementation of a reaction center-deficient *Rhodobacter sphaeroides pufLMX* deletion strain in trans with *pufBALM* does not restore the photosynthesis-positive phenotype. J. Bacteriol..

[19] Farchaus J.W., Barz W.P., Grunberg H., Oesterhelt D. (1992). Studies on the expression of the PufX polypeptide and its requirement for photoheterotrophic growth in *Rhodobacter sphaeroides*. EMBO J..

[20] Lilburn T.G., Haith C.E., Prince R.C., Beatty J.T. (1992). Pleiotropic effects of Pufx gene deletion on the structure and function of the photosynthetic apparatus of *Rhodobacter-capsulatus*. Biochim. Biophys. Acta.

[21] Comayras F., Jungas C., Lavergne J. (2005). Functional consequences of the organization of the photosynthetic apparatus in *Rhodobacter sphaeroides*. J. Biol. Chem..

[22] Walz T., Jamieson S.J., Bowers C.M., Bullough P.A., Hunter C.N. (1998). Projection structures of three photosynthetic complexes from *Rhodobacter sphaeroides*: LH2 at 6 Å, LH1 and RC-LH1 at 25 Å. J. Mol. Biol..

[23] McGlynn P., Hunter C.N., Jones M.R. (1994). The *Rhodobacter sphaeroides* PufX protein is not required for photosynthetic competence in the absence of a light harvesting system. FEBS Lett..

[24] Barz W.P., Oesterhelt D. (1994). Photosynthetic deficiency of a *pufX* deletion mutant of *Rhodobacter sphaeroides* is suppressed by point mutations in the light-harvesting complex genes *pufB* and *pufA*. Biochemistry.

[25] Lilburn T.G., Prince R.C., Beatty J.T. (1995). Mutation of the Ser2 codon of the light-harvesting B870 polypeptide of *Rhodobacter capsulatus* partially suppresses the pufX phenotype. J. Bacteriol..

[26] McGlynn P., Westerhuis W.H.J., Jones M.R., Hunter C.N. (1996). Consequences for the organisation of reaction centre/LH1 complexes of *Rhodobacter sphaeroides* arising from deletion of amino acid residues from the C terminus of the LH1α polypeptide. J. Biol. Chem..

[27] Olsen J.D., Martin E.C., Hunter C.N. (2017). The PufX quinone channel enables the LH1 antenna to bind more carotenoids for light collection and photoprotection. FEBS Lett..

[28] Hsin J., Gumbart J., Trabuco L.G., Villa E., Qian P., Hunter C.N., Schulten K. (2009). Protein-induced membrane curvature investigated through molecular dynamics flexible fitting. Biophys. J..

[29] Siebert C.A., Qian P., Fotiadis D., Engel A., Hunter C.N., Bullough P. (2004). The role of PufX in the molecular architecture of photosynthetic membranes in *Rhodobacter sphaeroides*. EMBO J..

[30] Adams P.G., Mothersole D.J., Ng I.W., Olsen J.D., Hunter C.N. (2011). Monomeric RC-LH1 core complexes retard LH2 assembly and intracytoplasmic membrane formation in PufX-minus mutants of *Rhodobacter sphaeroides*. Biochim. Biophys. Acta.

[31] Bahatyrova S., Frese R.N., Siebert C.A., Olsen J.D., van der Werf K.O., van Grondelle R., Niederman R.A., Bullough P.A., Otto C., Hunter C.N. (2004). The native architecture of a photosynthetic membrane. Nature.

[32] Frese R.N., Siebert C.A., Niederman R.A., Hunter C.N., Otto C., van Grondelle R. (2004). The long-range organization of a native photosynthetic membrane. Proc. Natl. Acad. Sci. U. S. A..

[33] Adams P.G., Hunter C.N. (2012). Adaptation of intracytoplasmic membranes to altered light intensity in *Rhodobacter sphaeroides*. Biochim. Biophys. Acta.

[34] Kumar S., Cartron M.L., Mullin N., Qian P., Leggett G.L., Hunter C.N., Hobbs J.K. (2016). Direct imaging of protein organisation in an intact bacterial organelle using high-resolution atomic force microscopy. ACS Nano.

[35] Frese R.N., Pàmies J.C., Olsen J.D., Bahatyrova S., van der Weij-de Wit C.D., Aartsma T.J., Otto C., Hunter C.N., Frenkel D., van Grondelle R. (2008). Protein shape and crowding drive domain formation and curvature in biological membranes. Biophys. J..

[36] Francia F., Wang J., Zischka H., Venturoli G., Oesterhelt D. (2002). Role of the N- and C-terminal regions of the PufX protein in the structural organization of the photosynthetic core complex of *Rhodobacter sphaeroides*. Eur. J. Biochem..

[37] Chi S.C., Mothersole D.J., Dilbeck P., Niedzwiedzki D.M., Zhang H., Qian P., Vasilev C., Grayson K.J., Jackson P.J., Martin E.C., Li Y., Holten D., Hunter C.N. (2015). Assembly of functional photosystem complexes in *Rhodobacter sphaeroides* incorporating carotenoids from the spirilloxanthin pathway. BBA Bioenerg..

[38] Jap B.K., Zulauf M., Scheybani T., Hefti A., Baumeister W., Aebi U., Engel A. (1992). 2D crystallization: from art to science. Ultramicroscopy.

[39] Tang G., Peng L., Baldwin P.R., Mann D.S., Jiang W., Rees I., Ludtke S.J. (2007). EMAN2: an extensible image processing suite for electron microscopy. J. Struct. Biol..

[40] Roy A., Kucukural A., Zhang Y. (2010). I-TASSER: a unified platform for automated protein structure and function prediction. Nat. Protoc..

[41] Crouch L.I., Holden-Dye K., Jones M.R. (2010). Dimerisation of the *Rhodobacter sphaeroides* RC-LH1 photosynthetic complex is not facilitated by a GxxxG motif in the PufX polypeptide. Biochim. Biophys. Acta.

[42] Fotiadis D., Qian P., Philippsen A., Bullough P.A., Engel A., Hunter C.N. (2004). Structural analysis of the RC-LH1 photosynthetic core complex of *Rhodospirillum rubrum* using atomic force microscopy. J. Biol. Chem..

[43] Pettersen E.F., Goddard T.D., Huang C.C., Couch G.S., Greenblatt D.M., Meng E.C., Ferrin T.E. (2004). UCSF Chimera—a visualization system for exploratory research and analysis. J. Comput. Chem..

[44] Jamieson S.J., Wang P., Qian P., Kirkland J.Y., Conroy M.J., Hunter C.N., Bullough P.A. (2002). Projection structure of the photosynthetic reaction centre-antenna complex of *Rhodospirillum rubrum* at 8.5 Å resolution. EMBO J..

[45] Niwa S., Yu L.J., Takeda K., Hirano Y., Kawakami T., Wang-Otomo Z.Y., Miki K. (2014). Structure of the LH1-RC complex from *Thermochromatium tepidum* at 3.0 Å. Nature.

[46] Majumder E.L.W., Olsen J.D., Qian P., Collins A.M., Hunter C.N., Blankenship R.E. (2016). Supramolecular organization of photosynthetic complexes in membranes of *Roseiflexus castenholzii*. Photosynth. Res..

[47] Collins A.M., Qian P., Tang Q., Bocian D.F., Hunter C.N., Blankenship R.E. (2010). The light-harvesting antenna system from the phototrophic bacterium *Roseiflexus castenholzii*. Biochemistry.

[48] Boonstra A.F., Visschers R.W., Calkoen F., van Grondelle R., van Bruggen E.J., Boekema E.J. (1993). Structural characterization of the B800-850 and B875 light-harvesting antenna complexes from *Rhodobacter sphaeroides* by electron-microscopy. Biochim. Biophys. Acta.

[49] Recchia P.A., Davis C.M., Lilburn T.G., Beatty J.T., Parkes-Loach P.S., Hunter C.N., Loach P.A. (1998). Isolation of the PufX protein from *Rhodobacter capsulatus* and *Rhodobacter sphaeroides*: evidence for its interaction with the alpha-polypeptide of the core light-harvesting complex. Biochemistry.

[50] Olsen J.D., Adams P.G., Hunter C.N. (2014). Aberrant assembly intermediates of the RC-LH1-PufX core complex of *Rhodobacter sphaeroides* imaged by atomic force microscopy. J. Biol. Chem..

[51] Mothersole D.J., Jackson P.J., Vasilev C., Tucker J.D., Brindley A.A., Dickman M.J., Hunter C.N. (2016). PucC and LhaA direct efficient assembly of the light-harvesting complexes in *Rhodobacter sphaeroides*. Mol. Microbiol..

[52] Westerhuis W.H.J., Sturgis J.N., Ratcliffe E.C., Hunter C.N., Niederman R.A. (2002). Isolation, size estimates and spectral heterogeneity of an oligomeric series of light-harvesting 1 complexes from *Rhodobacter sphaeroides*. Biochemistry.

