# Supplementary material for: The C-terminus of PufX plays a key role in dimerisation and assembly of the reaction center light-harvesting 1 complex from Rhodobacter sphaeroides
Source: Biochim Biophys Acta. 2017 Sep;1858(9):795–803. doi: 10.1016/j.bbabio.2017.06.001 (PMC5538271; doi:10.1016/j.bbabio.2017.06.001)
Supplement: Supplementary file 2 — Supplementary figures [file mmc1.pdf]

## Supplementary data

## S. 1  Photosynthetic growth curves.

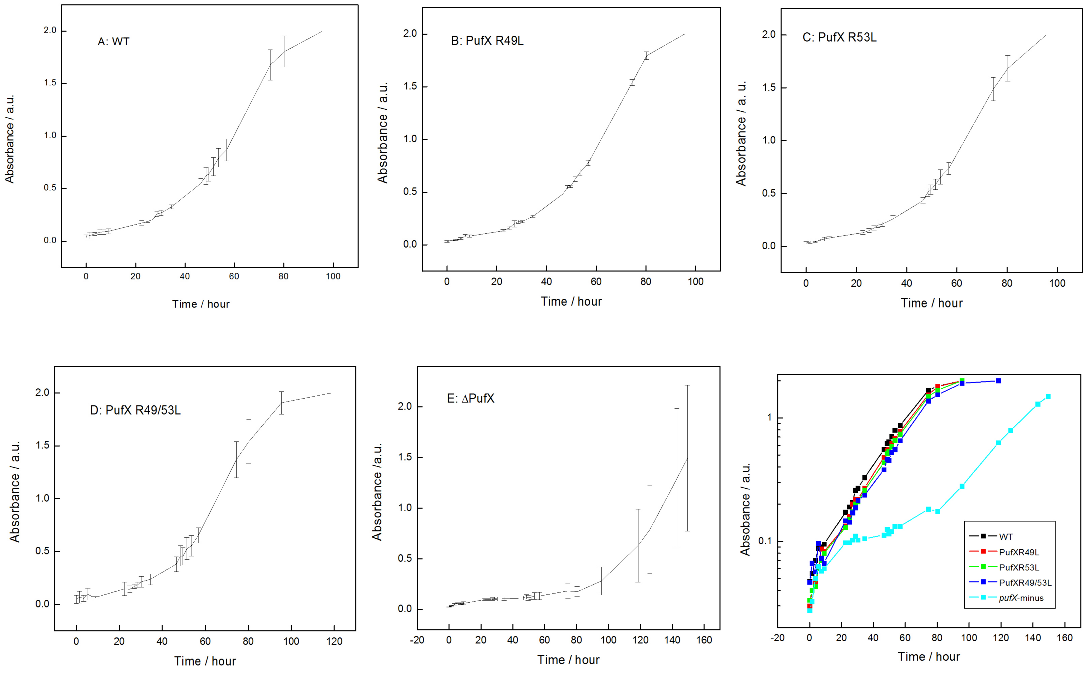


Photosynthetic growth curves of five different *Rba. sphaeroides* strains, each performed with three biological replicates. The panel (bottom right) displays the growth data for all mutants in semilogarithmic plots.

**S. 2 SDS-PAGE of purified core complexes**

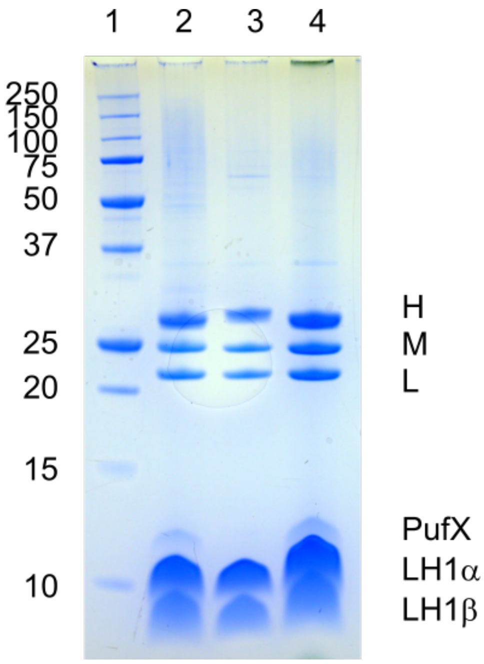


Lane 1 - Protein markers.
Lane 2 - wild-type core complex.
Lane 3 - PufX$^{-}$ core complexes.
Lane 4 - B, PufX R49/53L core complex.

**S. 3 Resolution estimation on 3D reconstruction models of the RC-LH1 core complexes from PufX⁻ and PufX R49/53L mutants of *Rba. sphaeroides***

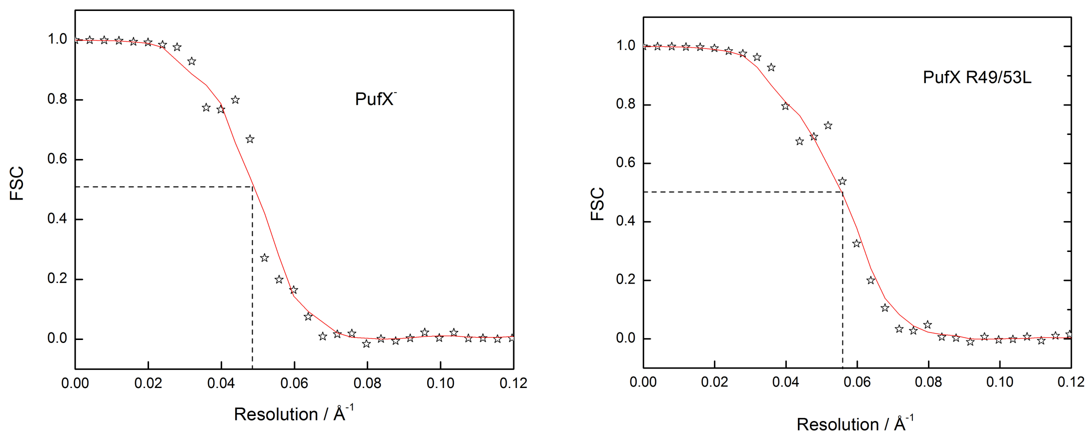


0.5 Fourier shell correlation criteria for resolution determination of core complexes. (Left) PufX⁻ mutant, 18.2 Å. (Right) PufX R49/53L mutant, 20.8 Å. The whole set of single particle data was separated into two, and were calculated independently to produce two 3D maps. Fourier shell correlations were calculated by comparison of the two maps in Fourier space against the resolution cutoff. 0.5 Fourier shell correlation was used for resolution estimation of the 3D reconstruction maps.

**S. 4 Prediction of the C-terminal conformations of mutant PufX structures**

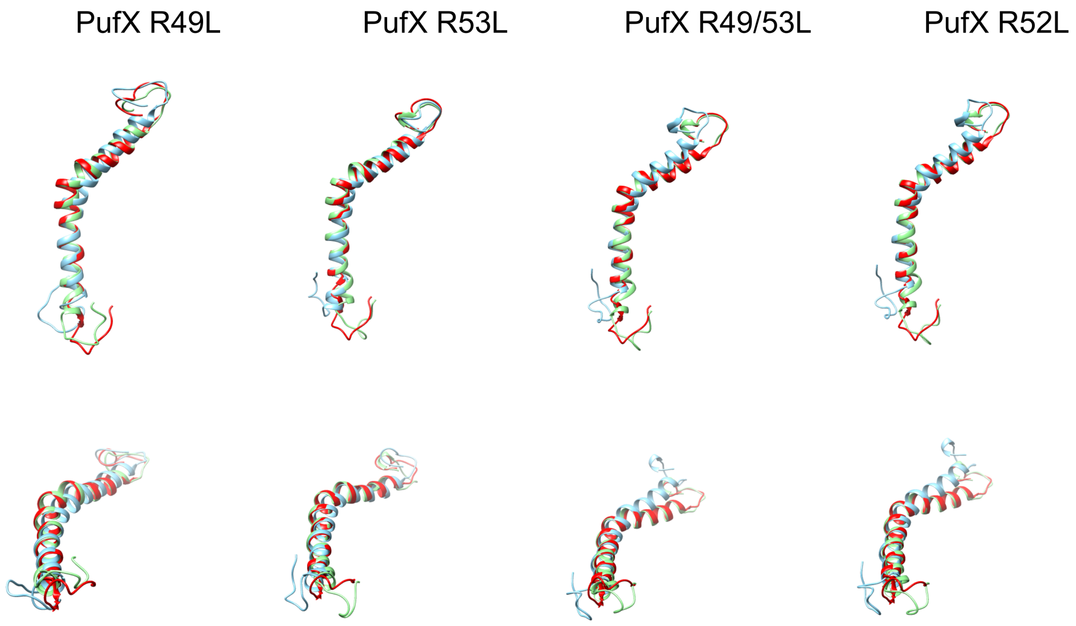


Predicted structures of PufX mutants were aligned against wild-type PufX (red); for each mutant PufX, the model in green has the lower RMSD value, and the cyan model a slightly higher value. The RMSD values (Å) are as follows: R49L – 2.75, 3.46; R53L – 3.45, 6.85; R49/53L – 3.10, 9.56; G52L – 2.80, 8.68. The PufX polypeptides are viewed in the plane of the membrane (top row), or are tilted to show the C-termini more clearly (bottom row).
